# Why Do Thin People Have Elevated All-Cause Mortality? Evidence on Confounding and Reverse Causality in the Association of Adiposity and COPD from the British Women’s Heart and Health Study

**DOI:** 10.1371/journal.pone.0115446

**Published:** 2015-04-17

**Authors:** Caroline Dale, Eveline Nüesch, David Prieto-Merino, Minkyoung Choi, Antoinette Amuzu, Shah Ebrahim, Juan P. Casas, George Davey-Smith

**Affiliations:** 1 Department of Non-communicable Disease Epidemiology, Faculty of Epidemiology and Population Health, London School of Hygiene & Tropical Medicine, London, United Kingdom; 2 Institute of Cardiovascular Science, University College London, London, United Kingdom; 3 CAiTE centre, School of Social and Community Medicine, University of Bristol, Bristol, United Kingdom; San Francisco Coordinating Center, UNITED STATES

## Abstract

Low adiposity has been linked to elevated mortality from several causes including respiratory disease. However, this could arise from confounding or reverse causality. We explore the association between two measures of adiposity (BMI and WHR) with COPD in the British Women’s Heart and Health Study including a detailed assessment of the potential for confounding and reverse causality for each adiposity measure. Low BMI was found to be associated with increased COPD risk while low WHR was not (OR = 2.2; 95% CI 1.3 – 3.1 versus OR = 1.2; 95% CI 0.7 – 1.6). Potential confounding variables (e.g. smoking) and markers of ill-health (e.g. unintentional weight loss) were found to be higher in low BMI but not in low WHR. Women with low BMI have a detrimental profile across a broad range of health markers compared to women with low WHR, and women with low WHR do not appear to have an elevated COPD risk, lending support to the hypothesis that WHR is a less confounded measure of adiposity than BMI. Low adiposity does not in itself appear to increase the risk of respiratory disease, and the apparent adverse consequences of low BMI may be due to reverse causation and confounding.

## Introduction

The epidemic of obesity that is sweeping high income countries and emerging in the more affluent sections of middle and low income countries is expected to increase the burden of chronic diseases and mortality. While there is little disagreement about the association between increased obesity and mortality, the causal relationship between low adiposity and mortality is much less clear. Determining the precise nature of the association between measures of adiposity and chronic diseases and hence the optimal level of adiposity is important for public health. A recent meta-analysis found lowest mortality risk at intermediate adiposity (Body Mass Index, BMI 25-<30; weight/height^2^) suggesting increased risk with lower adiposity [1]. Cause-specific analyses demonstrate that higher BMI is associated with higher risk of coronary heart disease (CHD), stroke and diabetes, whilst low BMI is associated with increased risk of respiratory disease, lung cancer and other smoking-related cancers [2]. Some commentators have suggested that the elevated respiratory disease mortality at low BMI may be causal [3].

The nature of the association of adiposity with chronic diseases is influenced by differences in the distribution of known confounders in different study populations, imperfect measurement of known confounders (residual confounding), imperfect modelling of non-linear confounders, as well as the distribution of unmeasured confounders. Inverse mortality associations at low BMI with COPD, lung cancer and upper aerodigestive cancer have been found to be stronger in smokers, suggesting residual confounding by smoking intensity is likely [2]. Apparent higher risk at low adiposity could also result from confounding due to underlying ill-health in study populations (“reverse causality”). Meta-analysis are often limited in their ability to make adjustments for confounders as they may not be measured in comparable ways or in sufficient detail in all studies, and therefore must find a compromise measurement that can fit across studies.

Different measures of adiposity such as waist circumference (WC) or waist-hip ratio (WHR) may have different associations with health outcomes compared to BMI. For example, in the context of cardiovascular disease WC and WHR might better capture central adiposity and therefore be stronger markers of risk [4–6] The European prospective study (EPIC) found less marked upturns with low mortality for WC or WHR than BMI [7], while the Scottish Health Survey found no upturn in mortality at low WHR [8]. Furthermore amongst older people increased mortality risk by WC has been shown within BMI categories.[9] It is unclear whether such differences between adiposity measures reflect real differences in risk or arise from differences in their associated confounding structures.

In this study we use data from the British Women’s Heart & Health Study (BWHHS) to explore in detail the nature of associations of two different measures of adiposity (BMI and WHR) with COPD including a detailed exploration of the association with potential confounders, biomarkers and markers of ill-health in order to highlight potential epidemiological pitfalls in the association between low BMI and elevated mortality.

## Materials and Methods

The BWHHS is a prospectively studied cohort study of 4,286 women aged between 60 and 79 at baseline from 23 towns across England, Wales and Scotland. Sampling procedure and data collection for the BWHHS is described in detail elsewhere.[10] Baseline measurements were taken in 1999–2000 with follow-up questionnaires in 2003, 2007 and 2010. Biomarkers were measured at baseline interview. Deaths and CVD events have been prospectively studied via the Data Linkage Service Health and Social Care Information Centre and biennial review of GP record. Ethical approval for the British Women’s Heart and Health Study was obtained from the London Medical Research and Ethics Committee (MREC) as well as the 23 local Research and Ethics Committee in each of the 23 towns participating in the study (S1 Appendix). Written informed consent was obtained from each participant in the study.

All variables included in these analyses were measured at baseline in 1999–2001.

### Measures of adiposity

Four approximately equally sized categories of BMI were derived for descriptive analyses: <24, 24–27, 27–30 and >30 kg/ m^2^. The smallest category was then further sub-divided into those with a BMI <22kg/ m^2^ in order to reflect the extreme of the BMI distribution amongst which all-cause mortality was found to be elevated by the Prospective Studies Collaboration.[2] 7.7% of women had BMI <22kg/ m^2^. WHR was derived from the average of two measures of waist circumference divided by the average of two measures of hip circumference, both measured in millimetres. WHR categories were derived in the same way as the BMI categories, i.e. division into quartiles with the smallest category subsequently further divided to include women with lowest adiposity as indexed by WHR. Therefore WHR categories were defined as: <0.72, 0.72–0.77, 0.77–0.81, 0.81–0.86 and >0.86. Women with BMI < 15 kg/m^2^ or >50 kg/ m^2^ and women with WHR <0.6 or > 1.1 were excluded from analyses.[2] Women with both measures of adiposity available were included in the analyses.

### Outcomes

COPD was defined according to the GOLD stage 2 criteria for moderate COPD;[11] when a woman’s FEV_1_ was found to be less than 70% of forced vital capacity (FVC), and forced expiratory volume in 1 second (FEV_1_) was less than 80% of the population predicted FEV_1_ adjusted for the age sex and height. Lung function tests were carried out using a digital meter Vitalograph. Women were required to perform a minimum of three reproducible FVC measures (within 5% of maximum FVC produced). The output that produced the highest sum of FVC and FEV1 were used in the analyses. Spirometric measurements fulfilled ATS/ERS requirements.

The MRC dyspnea scale was used as additional measure of respiratory function.[12][13] Women were considered to have symptoms of dyspnea if they reported usually bringing up phlegm in the morning on most days in the winter for as much as 3 months and having had a period of increased cough and phlegm in the past four years lasting for 3 weeks or more.

### Confounders—smoking

Smoking behaviour was measured as never versus ex/current smokers (self-reports), and amongst self-declared current smokers only cigarettes per day and plasma cotinine (ng/ml).

### Confounders—other lifestyle

Life-course socioeconomic position (SEP) was scored on a scale 0 to 10; where a higher score indicates more measures of socioeconomic deprivation across the life course as described previously.[14] Physical activity was measured as hours of moderate or vigorous activity per week (excluding housework) based on questions from the EPIC Norfolk questionnaire,[15] and categorised into two hours per week or less versus more than two hours per week. Healthy diet describes at least three healthy diet choices (brown or wholemeal bread; soft margarine or low calorie spreading fats; vegetable oil or olive oil cooking fats; semi-skimmed or skimmed milk).

### Confounders—markers of ill-health

Unintentional weight loss is self-reported unintentional weight change in the last 4 years. Locomotor disability is defined as difficulty with any one of: going up or down stairs, bending down, straightening up, keeping balance, going out of the house, walking 400 yards, and/or three or more falls in the past 12 months.[16] EQ-5D is a standardized instrument for measuring health-related quality of life;[17] EQ-5D utilities were calculated and the lowest third was considered to be low HR-QoL. Self-reported health was recorded and dichotomised into poor versus fair good or excellent. Self-reported number of medications was dichotomised into those taking multiple medications (two or more) versus only one or none.

### Biomarkers

All biomarkers were measured from venous blood samples taken at baseline; details of measurement procedures are reported elsewhere.[18, 19] Individual standardized summary scores for inflammation and coagulation biomarkers were calculated as previously described in the BWHHS, where higher scores indicate a higher load.[20]

## Statistical analyses

### Descriptive analyses with measures of adiposity

For each BMI and WHR category we estimated means (with 95% confidence intervals) for continuous outcomes and confounders, and percentages (with 95% confidence intervals) for binary outcomes and confounders using linear or logistic regression models. The goodness of fit was assessed by residual plots; where logarithmic transformation improved the model fit geometric means were calculated. Parameters were estimated using generalised estimating equation (GEE) to adjust the variances for clustering of women within GP practices in 23 towns.

### Stratified and adjusted analyses

Age-adjusted mean COPD prevalence by anthropometric category was stratified by smoking status (ever versus never). Then in a separate series of models mean COPD prevalence by anthropometric categories was adjusted by introducing age, lifestyle and socio-economic variables (SEP score, physical activity), markers of ill-health (unintended weight loss, locomotor disability, medications and EQ5D score) incrementally. Continuous confounding variables were centered at their respective means prior to inclusion in the adjusted models. For binary confounding variables COPD risk in the baseline category was modelled.

## Results

S1 Table shows the number and prevalence (binary variables) or mean and standard deviation (continuous variables) for all variables. Of the 3906 women with available data, 717 (18.4%) were classified as having moderate COPD according to the GOLD criteria. S2 Table shows the distribution and categorisation of BMI and WHR in the BWHHS. S3 Table shows the cross-tabulation of BMI and WHR categories. While many women were consistently extreme for both measures, some were extreme for one measure but not the other, especially at the lower end of the distributions; for example, only 21% of women with BMI <22 kg/ m^2^ also have WHR <0.72. The correlation coefficient for the two anthropometric measures is 0.37 (95% CI 0.35–0.40). S4 Table shows the distribution of all variables according to COPD diagnosis status.

### Descriptive association with low BMI and WHR

**Fig 1:**
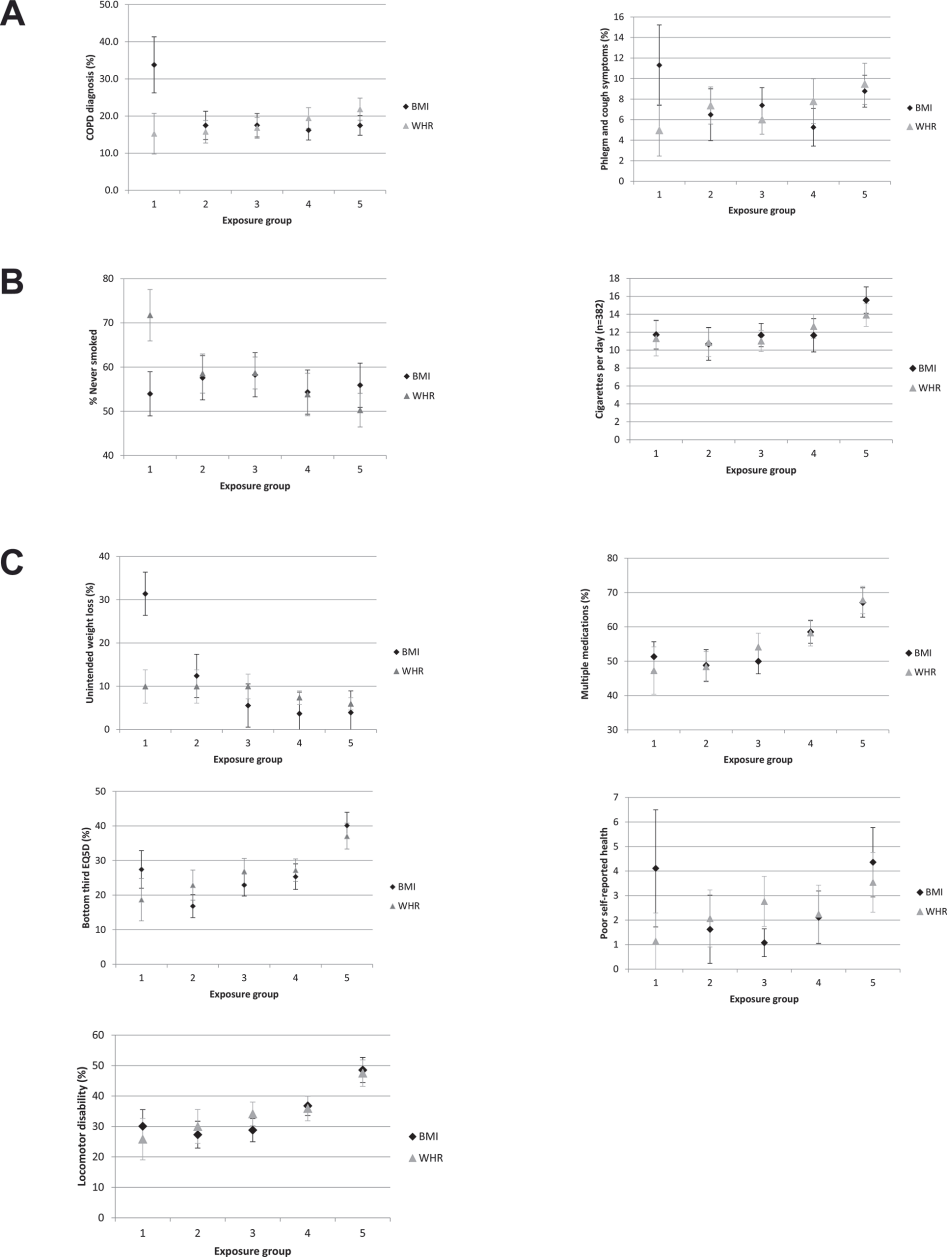
(A) Prevalence of COPD and symptoms of lung function by categories of BMI and WHR (lowest to highest). (B) Prevalence of smoking measures by categories of BMI and WHR (lowest to highest). (C) Prevalence of markers of ill-health by categories of BMI and WHR (lowest to highest).

For some measures, there was evidence for a difference in prevalence in the lowest BMI and WHR exposure categories (COPD, dyspnea symptoms, % never smoked and unintended weight loss) (Fig 1, S5 Table, S6 Table and S7 Table). COPD prevalence was higher amongst those with the lowest BMI compared to those with lowest WHR (15.2%; 95% CI 9.8–20.6 amongst those with a WHR<0.72 compared to 33.6%; 95% CI 26.1–41.1 amongst those with a BMI < 22kg/m^2^) (Fig 1A). Results for dyspnea symptoms were consistent with this.

Women with the lowest BMI were less likely to be never smokers than women with lowest WHR (WHR: 71.7%; 95% CI 65.9–77.6; BMI: 54.0%; 95% CI 46.4–61.5) (Fig 1B). Amongst self-declared smokers, plasma cotinine levels, an indicator of smoking intensity, were higher amongst women with low BMI (234.1 ng/ml; 95% CI 200.6–273.2) than low WHR (174.3 ng/ml; 95% CI 113.0–268.9). No clear differences were found between BMI and WHR for cigarettes per day as a measure of smoking intensity.

There was some weak evidence for low physical activity being more common amongst women with BMI<22kg/m^2^ compared to women with WHR<0.72 (20.0%; 95% CI 13.8–26.2 compared to 11.6%; 95% CI 8.1–15.0). Results also suggest healthy diet may be more common amongst those with low WHR compared to low BMI but it is not possible to rule out the role of chance in these findings. No clear evidence for differences in life-course SEP by BMI and WHR categories was observed (S1 Fig).

Of the measures of ill-health unintended weight loss was higher amongst those with the lowest BMI compared to those with lowest WHR (BMI: 31.4%; 95% CI 26.0–35.7 versus WHR: 9.9%; 95% CI 6.1–13.8). There was weak evidence for worse health amongst women with low BMI as more declared poor health compared to women with low WHR (4.1%; 95% CI 1.7–6.5 compared to 1.1%; 95% CI 0.0–2.4). Results were directionally consistent for multiple medications, low EQ5D and locomotor disability although no strong evidence was observed for these measures. Women in the lowest BMI category are as likely to report low health-related quality of life as women with a BMI>27.

Inflammation and coagulation biomaker scores were both directionally higher for BMI in the lowest exposure group compared to WHR, although it is not possible to exclude the role of chance in these findings (S2 Fig).

In summary, women with low BMI are more likely to be diagnosed with COPD and report dyspnea symptoms. They are more likely to report a spectrum of measures of ill-health, including poor health and unintended weight, with weaker evidence for lower quality of life and taking more medications. In addition, they are more likely to report ever having smoked and have higher smoking intensity among those women who currently smoke. There is weaker evidence that women with low BMI may also have lower levels of physical activity and adverse inflammation and coagulation biomarker profiles.

### Adjustment

**Fig 2:**
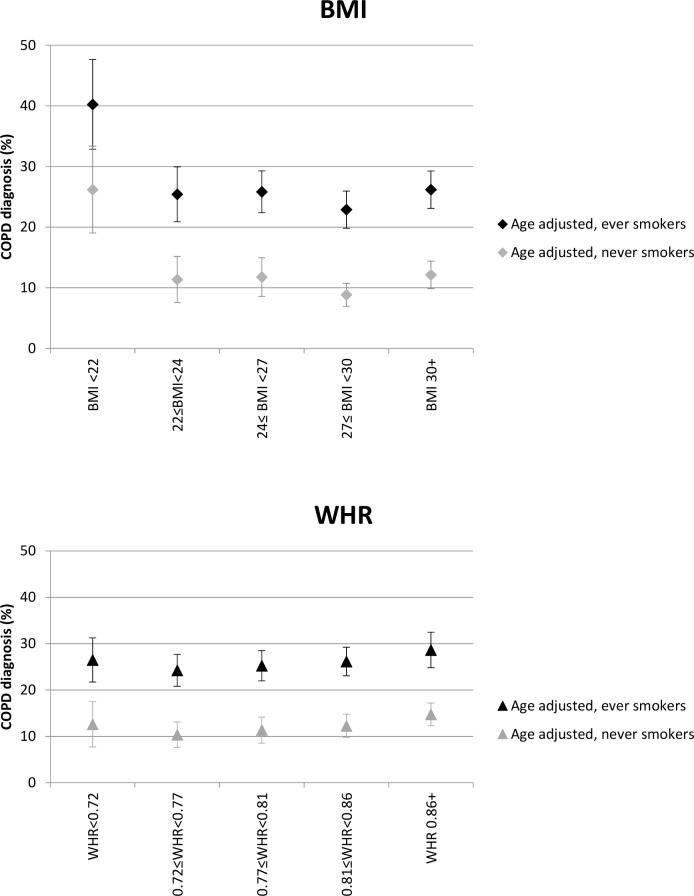
Prevalence of COPD adjusted for age and stratified by smoking status.

As expected, estimated age-adjusted prevalence of COPD was lower amongst never smokers in all anthropometric categories (Fig 2). Prevalence of COPD was elevated amongst never smokers in the lowest BMI group (26.1%; 95% CI 19.0–33.3) and is comparable to that of smokers with 22<BMI<24 (25.4%; 95% CI 20.9–29.9). Prevalence of COPD was clearly higher for smokers than non-smokers in all categories of WHR.

**Fig 3:**
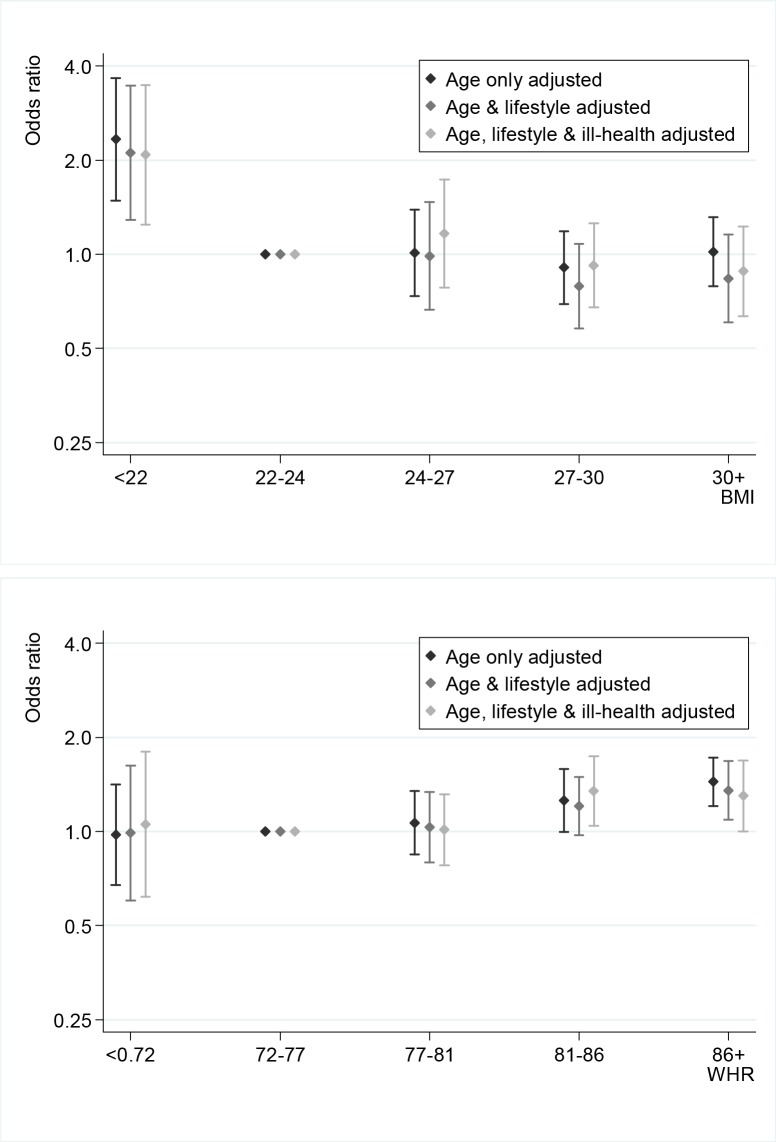
Gradual adjustment of associations between COPD and adiposity by age, lifestyle and ill-health.

The effect of the incremental adjustment on COPD diagnosis by BMI category relative to baseline 22<BMI<24 is illustrated in Fig 3 (S8 Table). The odds of COPD in the lowest BMI category remained higher (OR = 2.2; 95% CI 1.3–3.1) compared to normal BMI even after full adjustment for age, lifestyle and ill-health, although the point estimates did move closer to one on adjustment. There was no evidence for elevated odds of COPD for any category of higher BMI. Unlike BMI, there is no evidence for increased odds of COPD in the lowest WHR category relative to the baseline 0.72<WHR<0.77 (OR = 1.2; 95% CI 0.7–1.6), but we found increasing odds of COPD in the higher WHR categories.

## Discussion

Low BMI has previously been linked to elevated mortality from several causes including respiratory disease and liver disease. In this paper we explored the characteristics of women with low BMI and how they differ from women with higher BMI as well as women with low WHR. We found evidence to support a different shaped association with COPD for BMI compared to WHR, with higher COPD risk observed amongst women with low BMI. Furthermore, analysis of the distribution of several potential confounding variables suggested that these were distributed differently according to categories of BMI compared to categories of WHR, lending support to the hypothesis that low BMI—high mortality associations may be an artefact produced by confounding and reverse causation.

Low BMI can be the result of favourable behaviours (such as good diet and high physical activity) or it may a marker of underlying disease (wasting). Clearly each of these will be associated with very different characteristics and future health outcomes. Given the relatively older age of women in the BWHHS (in common with many prospective cohorts), we hypothesized that the majority of women with low BMI are likely to fall into the second category. In these analyses we find support for the hypothesis that women with low BMI have a detrimental profile across a broad range of health markers, including weight loss. Weight loss is a well-established predictor of mortality in older populations;[21, 22] accelerating three years before death for cancer and five years before cardiovascular deaths.[23] Weight loss can also be a symptom of COPD.[24]

Unlike low BMI, women who have very low WHR do not appear to have an elevated risk of COPD. Furthermore, low WHR appears to be less likely to be associated with the spectrum of adverse lifestyle (smoking, low physical activity) and markers of ill-health. These findings lend support to the hypothesis that WHR is a less confounded measure of adiposity than BMI. However, while results appear consistent across a broad range of measures, the relatively small number of women with extremely low BMI and WHR means our results are imprecise and require replication in other studies. Different shaped functions for the association between anthropometric measures and mortality have previously been reported, with less marked upturn at the lower end of the distribution for WHR and WC [7]. The Emerging Risk Factors collaboration also report a J-shaped association between BMI and coronary heart disease, but a linear association for WC and WHR.[25]

These findings have important implications for epidemiological studies seeking to reveal the true association between adiposity and disease outcomes. Conventional adjustment for variables such as smoking (current, ex-, never) is unlikely to be adequate to remove the residual confounding effects of smoking intensity.[26] We find that the association between low BMI and COPD is relatively unaffected by adjustment for the life-style variables and markers of ill-health available in the BWHHS with a strong association remaining. Again, this suggests that residual confounding, both from lifestyle factors and ill-health (“reverse causation”), may remain as important limitations in epidemiological studies which often adjust for far fewer variables. Furthermore, while studies may exclude the first years of follow-up (commonly five) this is unlikely to be sufficient to remove the potential effect of COPD on weight loss given that its onset is gradual and progressive over many years.[27] The problem of reverse causality in observational studies has recently been highlighted in a large Chinese study showing that even in participants “apparently free” from disease at baseline the mortality hazard ratio for low BMI is substantially stronger in those who die in the first five years [28]. Indirect markers of BMI (childhood BMI [29], offspring BMI[30], or genetic variants associated with adiposity[31]) may therefore be helpful to understand the nature of the associations of low BMI with the increased risk of some diseases since they minimise the potential for confounding due to ill health. In a large sample drawn from the Swedish population register, high offspring BMI was associated with increased risk of CVD, diabetes and some cancers, but low offspring BMI was not associated with increased risk of respiratory disease and lung cancer.[30] Mendelian randomization studies using multiple genetic instruments for BMI and methods for handling non-linearity would help to bring clarity to the association of low BMI with disease.

### Limitations

Data are drawn from an elderly population of mainly white British women. Consistent with other sources, BMI greater than 25kg/m^2^ in this population is high, at almost four-fifths.[32] However, rates of obesity have been increasing rapidly in recent years in the UK;[32] therefore this population of elderly women who lived the majority of their lives in a lower obesity setting may not reflect more contemporary British populations. Replication of results in other populations is therefore needed. 22kg/m^2^ was chosen as the cut-point for the category of women with lowest BMI on the basis of prior evidence from the Prospective Studies Collaboration.[2] However, choice of cut-points may influence results and appropriate cut-points may vary between different populations. BWHHS women have relatively low prevalence of smoking and physical activity (when housework is excluded).[33, 34] It is possible that differential associations according to anthropometric measure may be more marked in younger populations and those with higher background rates of smoking.

## Conclusions

Women with low BMI have a detrimental profile of health makers compared to women with low WHR. Unlike BMI, we find women who have extremely low WHR do not appear to have an elevated risk of COPD, lending some support to the hypothesis that WHR is a less confounded measure of adiposity than BMI. Results suggest conventional epidemiological studies seeking to reveal the true association between adiposity and disease outcome may be unable to adequately remove the effects of residual confounding or reverse causality.

## Supporting Information

S1 Table Distribution of all variables; mean and SD or percentage. (DOCX)

S2 Table Distribution and categorisation of BMI (kg/m2) and WHR in BWHHS. (DOCX)

S3 Table Bivariate distribution of women by anthropometric measures, percent. (DOCX)

S4 Table Distribution of all variables by COPD diagnosis; mean and SD or percentage. (DOCX)

S5 Table Distribution of COPD, respiratory function and symptoms by categories of BMI and WHR in BWHHS, % or mean (95% CIs). (DOCX)

S6 Table Distribution of smoking and other lifestyle variables by categories of BMI and WHR in BWHHS, % or mean (95% CIs). (DOCX)

S7 Table Distribution of markers of ill-health and biomarker scores by categories of BMI and WHR in BWHHS, % or mean (95% CIs). (DOCX)

S8 Table Incrementally adjusted odds ratios and 95% CIs of COPD by BMI and WHR categories. (DOCX)

S1 Fig SES and lifestyle confounders by BMI and WHR categories. (DOCX)

S2 Fig Mean & 95% CI biomarker scores by BMI and WHR categories. (DOCX)

S1 Appendix (DOCX)
