# Supplementary material for: Why Do Thin People Have Elevated All-Cause Mortality? Evidence on Confounding and Reverse Causality in the Association of Adiposity and COPD from the British Women’s Heart and Health Study
Source: PLoS One. 2015 Apr 17;10(4):e0115446. doi: 10.1371/journal.pone.0115446 (PMC4401726; doi:10.1371/journal.pone.0115446)
Supplement: S1 Table — (DOCX) [file pone.0115446.s001.docx]

**S1 Table. Distribution of all variables; mean and SD or percentage**

|  | Mean (%) | SD | N |
| --- | --- | --- | --- |
|  |  |  |  |
| BMI (kg/m^2^) | 27.6 | 4.9 | 3930 |
| WHR | 0.8 | 0.1 | 3930 |
|  |  |  |  |
| COPD GOLD stage 2 (%) | 18.4% |  | 3879 |
| FEV_1_ | 2.0 | 0.5 | 3885 |
| Phlegm or cough symptoms | 7.5% |  | 3692 |
|  |  |  |  |
| Age | 68.8 | 5.5 | 3930 |
| Never smoke (%) | 56.3% |  | 3928 |
| Cigarettes per day in smokers | 12.3 | 6.7 | 382 |
| Cotinine (ng/ml) | 28.0 | 86.4 | 3770 |
| Cotinine (ng/ml) in smokers | 228.6 | 131.8 | 414 |
| Lifecourse SES score (0-10) | 4.2 | 2.2 | 3264 |
| Low physical activity (< 2 hrs moderate or vigorous / wk) | 18.5% |  | 3772 |
| Healthy diet (%) | 56.4% |  | 3480 |
| Unintended weight loss (%) | 7.4% |  | 3739 |
| Multiple medications (%) | 57.0% |  | 3930 |
| Locomotor disability (%) | 36.5% |  | 3433 |
| Poor self-reported health (%) | 2.6% |  | 3721 |
| Low EQ5D score (%) | 28.1% |  | 3331 |
| Standardized inflammation biomarkers score | 4.9 | 2.6 | 3779 |
| Standardized coagulation biomarkers score | 4.8 | 1.6 | 3779 |
|  |  |  |  |
