# Supplementary material for: Why Do Thin People Have Elevated All-Cause Mortality? Evidence on Confounding and Reverse Causality in the Association of Adiposity and COPD from the British Women’s Heart and Health Study
Source: PLoS One. 2015 Apr 17;10(4):e0115446. doi: 10.1371/journal.pone.0115446 (PMC4401726; doi:10.1371/journal.pone.0115446)
Supplement: S2 Table — (DOCX) [file pone.0115446.s002.docx]

S2 Table. Distribution and categorisation of BMI (kg/m2) and WHR in BWHHS

| **Exposure groups** | **%** | **Mean (SD)** |
| --- | --- | --- |
|  |  |  |
| BMI <22 | 7.7 | 20.0 (1.2) |
| 22≤BMI<24 | 11.5 | 22.6 (0.5) |
| 24≤ BMI <27 | 27.1 | 25.0 (0.8) |
| 27≤ BMI <30 | 24.2 | 27.9 (0.8) |
| BMI 30+ | 29.5 | 33.5 (3.7) |
|  |  |  |
| WHR<0.72 | 7.0 | 0.70 (0.02) |
| 0.72≤WHR<0.77 | 18.0 | 0.75 (0.01) |
| 0.77≤WHR<0.81 | 25.1 | 0.79 (0.01) |
| 0.81≤WHR<0.86 | 25.0 | 0.84 (0.01) |
| WHR 0.86+ | 24.9 | 0.91 (0.04) |
