# Supplementary material for: Why Do Thin People Have Elevated All-Cause Mortality? Evidence on Confounding and Reverse Causality in the Association of Adiposity and COPD from the British Women’s Heart and Health Study
Source: PLoS One. 2015 Apr 17;10(4):e0115446. doi: 10.1371/journal.pone.0115446 (PMC4401726; doi:10.1371/journal.pone.0115446)
Supplement: S3 Table — (DOCX) [file pone.0115446.s003.docx]

S3 Table. Bivariate distribution of women by anthropometric measures, percent.

|  | WHR<0.72 | 0.72≤WHR<0.77 | 0.77≤WHR<0.81 | 0.81≤WHR<0.86 | WHR 0.86+ |  | Total |
| --- | --- | --- | --- | --- | --- | --- | --- |
|  |  |  |  |  |  |  |  |
| BMI <22 | 21 | 39 | 23 | 13 | 4 |  | 302 |
| 22≤BMI<24 | 14 | 28 | 33 | 17 | 9 |  | 451 |
| 24≤ BMI <27 | 7 | 23 | 30 | 25 | 16 |  | 1,067 |
| 27≤ BMI <30 | 4 | 13 | 24 | 29 | 30 |  | 952 |
| BMI 30+ | 3 | 9 | 20 | 28 | 41 |  | 1,158 |
|  |  |  |  |  |  |  |  |
| Total | 273 | 709 | 985 | 984 | 979 |  | 3,930 |
