# Supplementary material for: Why Do Thin People Have Elevated All-Cause Mortality? Evidence on Confounding and Reverse Causality in the Association of Adiposity and COPD from the British Women’s Heart and Health Study
Source: PLoS One. 2015 Apr 17;10(4):e0115446. doi: 10.1371/journal.pone.0115446 (PMC4401726; doi:10.1371/journal.pone.0115446)
Supplement: S4 Table — (DOCX) [file pone.0115446.s004.docx]

**S4 Table. Distribution of all variables by COPD diagnosis; mean and SD or percentage**

| **COPD Diagnosis** | **Yes** | | |  | **No** | | |
| --- | --- | --- | --- | --- | --- | --- | --- |
|  | **Mean (%)** | **SD** | **N** |  | **Mean (%)** | **SD** | **N** |
|  |  |  |  |  |  |  |  |
| BMI (kg/m^2^) | 27.0 | 5.2 | 715 |  | 27.7 | 4.8 | 3164 |
| WHR | 0.8 | 0.1 | 715 |  | 0.8 | 0.1 | 3164 |
|  |  |  |  |  |  |  |  |
| FEV_1_ | 1.4 | 0.4 | 715 |  | 2.1 | 0.4 | 3164 |
| Phlegm or cough symptoms | 17.5% |  | 664 |  | 5.3% |  | 2985 |
|  |  |  |  |  |  |  |  |
| Age | 69.7 | 5.4 | 715 |  | 68.6 | 5.5 | 3164 |
| Never smoke (%) | 37.6% |  | 715 |  | 60.4% |  | 3162 |
| Cigarettes per day | 12.6 | 7.3 | 162 |  | 11.3 | 6.7 | 251 |
| Cotinine (ng/ml) | 65.3 | 125.7 | 687 |  | 19.7 | 72.7 | 3033 |
| Lifecourse SES score (0-10) | 4.7 | 2.2 | 575 |  | 4.1 | 2.1 | 2651 |
| Low physical activity (< 2 hrs moderate or vigorous / wk) | 26.3% |  | 678 |  | 16.5% |  | 3053 |
| Healthy diet (%) | 50.4% |  | 629 |  | 57.9% |  | 2812 |
| Unintended weight loss (%) | 13.9% |  | 669 |  | 6.0% |  | 3028 |
| Multiple medications (%) | 66.6% |  | 715 |  | 55.0% |  | 3164 |
| Locomotor disability (%) | 45.9% |  | 623 |  | 34.4% |  | 2773 |
| Poor self-reported health (%) | 3.7% |  | 673 |  | 2.2% |  | 3005 |
| Low EQ5D score (%) | 31.8% |  | 576 |  | 27.2% |  | 2716 |
| Standardized inflammation biomarkers score | 5.6 | 2.5 | 692 |  | 4.8 | 2.6 | 3037 |
| Standardized coagulation biomarkers score | 5.0 | 1.6 | 692 |  | 4.7 | 1.5 | 3037 |
|  |  |  |  |  |  |  |  |
