# Supplementary material for: Why Do Thin People Have Elevated All-Cause Mortality? Evidence on Confounding and Reverse Causality in the Association of Adiposity and COPD from the British Women’s Heart and Health Study
Source: PLoS One. 2015 Apr 17;10(4):e0115446. doi: 10.1371/journal.pone.0115446 (PMC4401726; doi:10.1371/journal.pone.0115446)
Supplement: S5 Table — (DOCX) [file pone.0115446.s005.docx]

S5 Table. Distribution of COPD, respiratory function and symptoms by categories of BMI and WHR in BWHHS, % or mean (95% CIs)

|  | COPD (%) | | |  | FEV_1_ | | |  | Phlegm & cough symptoms | | |
| --- | --- | --- | --- | --- | --- | --- | --- | --- | --- | --- | --- |
|  |  |  |  |  |  |  |  |  |  |  |  |
| BMI <22 | 33.8 | (26.2 - | 41.3) |  | 1.8 | (1.7 - | 1.9) |  | 11.3 | (7.4 - | 15.2) |
| 22≤BMI<24 | 17.5 | (13.7 - | 21.3) |  | 2.0 | (2.0 - | 2.1) |  | 6.5 | (4.0 - | 9.0) |
| 24≤ BMI <27 | 17.5 | (14.4 - | 20.7) |  | 2.0 | (2.0 - | 2.1) |  | 7.4 | (5.7 - | 9.1) |
| 27≤ BMI <30 | 16.2 | (13.5 - | 18.8) |  | 2.0 | (1.9 - | 2.0) |  | 5.3 | (3.4 - | 7.1) |
| BMI 30+ | 17.5 | (14.8 - | 20.1) |  | 1.9 | (1.9 - | 2.0) |  | 8.8 | (7.2 - | 10.3) |
|  |  |  |  |  |  |  |  |  |  |  |  |
| WHR<0.72 | 15.2 | (9.8 - | 20.7) |  | 2.13 | (2.0 - | 2.2) |  | 5.0 | (2.5 - | 7.5) |
| 0.72≤WHR<0.77 | 15.8 | (12.7 - | 18.8) |  | 2.05 | (2.0 - | 2.1) |  | 7.4 | (5.6 - | 9.2) |
| 0.77≤WHR<0.81 | 16.8 | (14.0 - | 19.6) |  | 2.02 | (2.0 - | 2.1) |  | 6.0 | (4.6 - | 7.4) |
| 0.81≤WHR<0.86 | 19.4 | (16.6 - | 22.3) |  | 1.94 | (1.9 - | 2.0) |  | 7.8 | (5.6 - | 10.0) |
| WHR 0.86+ | 21.8 | (18.9 - | 24.8) |  | 1.89 | (1.8 - | 1.9) |  | 9.5 | (7.5 - | 11.5) |
|  |  |  |  |  |  |  |  |  |  |  |  |
