# Supplementary material for: Why Do Thin People Have Elevated All-Cause Mortality? Evidence on Confounding and Reverse Causality in the Association of Adiposity and COPD from the British Women’s Heart and Health Study
Source: PLoS One. 2015 Apr 17;10(4):e0115446. doi: 10.1371/journal.pone.0115446 (PMC4401726; doi:10.1371/journal.pone.0115446)
Supplement: S6 Table — (DOCX) [file pone.0115446.s006.docx]

S6 Table. Distribution of smoking and other lifestyle variables by categories of BMI and WHR in BWHHS, % or mean (95% CIs)

|  |  | Never smoke (%) | Cotinine in smoker^#+^ (ng/ml) | Cigs. Per day (smoker) |  | SEP lifecourse score | <2 hrs physical activity per wk (%) | Healthy diet (%) |
| --- | --- | --- | --- | --- | --- | --- | --- | --- |
| BMI <22 |  | 53.95 [46.38;61.51] | 234.14 [200.80;273.03] | 11.73 [10.12;13.33] |  | 4.2 [3.8;4.7] | 20.00 [13.78;26.22] | 47.3 [40.0,54.5] |
| 22≤BMI<24 |  | 57.58 [53.82;61.34] | 164.14 [119.75;224.99] | 10.69 [8.86;12.53] |  | 3.8 [3.6;4.1] | 14.67 [10.47;18.88] | 53.4 [46.8,59.9] |
| 24≤ BMI <27 |  | 58.26 [53.87;62.65] | 169.45 [147.27;194.97] | 11.68 [10.37;12.98] |  | 4.0 [3.7;4.3] | 16.45 [13.40,19.52] | 57.1 [52.6,61.6] |
| 27≤ BMI <30 |  | 54.35 [50.61;58.08] | 146.4 [109.93;194.98] | 11.66 [9.79;13.52] |  | 4.2 [3.8;4.5] | 19.98 [16.99,22.97] | 59.6 [55.1,64.2] |
| BMI 30+ |  | 55.90 [52.66;59.14] | 144.13 [103.61;200.49] | 15.58 [14.11;17.05] |  | 4.6 [4.3;4.9] | 20.29 [18.00,22.57] | 56.9 [53.4,60.4] |
|  |  |  |  |  |  |  |  |  |
| WHR<0.72 |  | 71.74 [65.92;77.56] | 174.32 [113.25;268.34] | 10.63 [8.98;12.27] |  | 4.0 [3.7;4.3] | 11.57 [8.12;15.01] | 55.6 [49.4,61.9] |
| 0.72≤WHR<0.77 |  | 58.53 [54.1;62.96] | 177.93 [136.86;231.31] | 10.29 [8.69;11.88] |  | 3.9 [3.6;4.3] | 14.76 [11.53;17.99] | 58.7 [54.4,63.0] |
| 0.77≤WHR<0.81 |  | 58.66 [55.05;62.28] | 155.8 [123.66;196.29] | 10.68 [9.30;12.06] |  | 4.3 [4.0;4.6] | 16.77 [13.06;20.48] | 55.3 [51.6,59.0] |
| 0.81≤WHR<0.86 |  | 53.81 [49.00;58.62] | 164.65 [125.82;215.46] | 11.71 [10.32;13.09] |  | 4.3 [3.9;4.6] | 20.32 [17.41;23.22] | 55.4 [51.1,59.6] |
| WHR 0.86+ |  | 50.25 [46.45;54.06] | 160.85 [131.05;197.44] | 13.73 [12.55;14.91] |  | 4.4 [4.0;4.8] | 23.04 [20.25;25.82] | 57.2 [53.5,60.9] |
