# Supplementary material for: Why Do Thin People Have Elevated All-Cause Mortality? Evidence on Confounding and Reverse Causality in the Association of Adiposity and COPD from the British Women’s Heart and Health Study
Source: PLoS One. 2015 Apr 17;10(4):e0115446. doi: 10.1371/journal.pone.0115446 (PMC4401726; doi:10.1371/journal.pone.0115446)
Supplement: S7 Table — (DOCX) [file pone.0115446.s007.docx]

S7 Table. Distribution of markers of ill-health and biomarker scores by categories of BMI and WHR in BWHHS, % or mean (95% CIs)

|  | Unintended weight loss (%) | Locomotor disability (%) | Bottom third EQ5D (%) | Poor health (%) | Multiple medications (%) |
| --- | --- | --- | --- | --- | --- |
| BMI <22 | 31.38 [26.03;36.73] | 29.96 [24.58;35.35] | 27.45 [22.01;32.89] | 4.1 [1.7;6.5] | 51.3 [47.0;55.7] |
| 22≤BMI<24 | 12.39 [9.42;15.35] | 27.16 [22.75;31.57] | 16.83 [13.52;20.16] | 1.6 [0.2;3.0] | 48.8 [44.2;53.4] |
| 24≤ BMI <27 | 5.53 [4.14;6.92] | 29.08 [25.18;32.98] | 22.92 [19.75,26.09] | 1.1 [0.5;1.6] | 50.0 [46.3;53.6] |
| 27≤ BMI <30 | 3.65 [2.23;5.06] | 36.87 [33.76;39.99] | 25.34 [21.64,29.04] | 2.1 [1.0;3.2] | 58.5 [55.2;61.8] |
| BMI 30+ | 3.91 [2.59;5.22] | 48.98 [44.88;53.08] | 40.14 [36.31,43.98] | 4.4 [2.9;5.8] | 67.1 [62.8;71.4] |
|  |  |  |  |  |  |
| WHR<0.72 | 9.92 [6.07;13.77] | 26.03 [19.34;32.73] | 18.72 [12.58;24.87] | 1.1 [0.0;2.4] | 47.3 [40.3;54.2] |
| 0.72≤WHR<0.77 | 9.94 [7.09;12.8] | 30.11 [24.53;35.69] | 22.9 [18.54;27.27] | 2.1 [0.9;3.2] | 48.4 [44.0;52.8] |
| 0.77≤WHR<0.81 | 7.36 [5.77;8.94] | 34.11 [30.25;37.97] | 26.9 [23.21;30.59] | 2.8 [1.7;3.8] | 54.1 [50.1;58.2] |
| 0.81≤WHR<0.86 | 5.93 [4.51;7.35] | 36.07 [32.02;40.12] | 27.24 [24.01;30.47] | 2.2 [1.1;3.4] | 58.2 [54.4;62.0] |
| WHR 0.86+ | 6.49 [4.5;8.48] | 47.82 [43.53;52.11] | 37.06 [33.32;40.81] | 3.5 [2.3;4.8] | 67.8 [63.8;71.8] |
