# Supplementary material for: Why Do Thin People Have Elevated All-Cause Mortality? Evidence on Confounding and Reverse Causality in the Association of Adiposity and COPD from the British Women’s Heart and Health Study
Source: PLoS One. 2015 Apr 17;10(4):e0115446. doi: 10.1371/journal.pone.0115446 (PMC4401726; doi:10.1371/journal.pone.0115446)
Supplement: S8 Table — (DOCX) [file pone.0115446.s008.docx]

**S8 Table. Incrementally adjusted odds ratios and 95% CIs of COPD by BMI and WHR categories**

| Adjustment | Age only | | |  | Age & lifestyle | | |  | Age, lifestyle & ill-health | | |  | Age, lifestyle, ill-health & biomarkers | | |
| --- | --- | --- | --- | --- | --- | --- | --- | --- | --- | --- | --- | --- | --- | --- | --- |
|  |  |  |  |  |  |  |  |  |  |  |  |  |  |  |  |
| BMI <22 | 2.3 | (1.5 - | 3.7) |  | 2.1 | (1.3 - | 3.5) |  | 2.1 | (1.2 - | 3.5) |  | 2.2 | (1.3 - | 3.7) |
| 22≤BMI<24* | 1 |  |  |  | 1 |  |  |  | 1 |  |  |  | 1 |  |  |
| 24≤ BMI <27 | 1.0 | (0.7 - | 1.4) |  | 1.0 | (0.7 - | 1.5) |  | 1.2 | (0.8 - | 1.7) |  | 1.1 | (0.7 - | 1.7) |
| 27≤ BMI <30 | 0.9 | (0.7 - | 1.2) |  | 0.8 | (0.6 - | 1.1) |  | 0.9 | (0.7 - | 1.3) |  | 0.8 | (0.6 - | 1.1) |
| BMI 30+ | 1.0 | (0.8 - | 1.3) |  | 0.8 | (0.6 - | 1.2) |  | 0.9 | (0.6 - | 1.2) |  | 0.7 | (0.5 - | 1.0) |
|  |  |  |  |  |  |  |  |  |  |  |  |  |  |  |  |
| WHR<0.72 | 1.0 | (0.7 - | 1.4) |  | 1.0 | (0.6 - | 1.6) |  | 1.1 | (0.6 - | 1.8) |  | 1.2 | (0.7 - | 2.0) |
| 0.72≤WHR<0.77* | 1 |  |  |  | 1 |  |  |  | 1 |  |  |  | 1 |  |  |
| 0.77≤WHR<0.81 | 1.1 | (0.8 - | 1.4) |  | 1.0 | (0.8 - | 1.3) |  | 1.0 | (0.8 - | 1.3) |  | 1.0 | (0.8 - | 1.3) |
| 0.81≤WHR<0.86 | 1.3 | (1.0 - | 1.6) |  | 1.2 | (1.0 - | 1.5) |  | 1.3 | (1.0 - | 1.7) |  | 1.2 | (0.9 - | 1.6) |
| WHR 0.86+ | 1.4 | (1.2 - | 1.7) |  | 1.4 | (1.1 - | 1.7) |  | 1.3 | (1.0 - | 1.7) |  | 1.1 | (0.8 - | 1.6) |
|  |  |  |  |  |  |  |  |  |  |  |  |  |  |  |  |
| * Baseline group |  |  |  |  |  |  |  |  |  |  |  |  |  |  |  |
