# Supplementary material for: Why Do Thin People Have Elevated All-Cause Mortality? Evidence on Confounding and Reverse Causality in the Association of Adiposity and COPD from the British Women’s Heart and Health Study
Source: PLoS One. 2015 Apr 17;10(4):e0115446. doi: 10.1371/journal.pone.0115446 (PMC4401726; doi:10.1371/journal.pone.0115446)
Supplement: S2 Fig — (DOCX) [file pone.0115446.s010.docx]

**S2 Fig. Mean & 95% CI biomarker scores by BMI and WHR categories**
