# Supplementary material for: Why Do Thin People Have Elevated All-Cause Mortality? Evidence on Confounding and Reverse Causality in the Association of Adiposity and COPD from the British Women’s Heart and Health Study
Source: PLoS One. 2015 Apr 17;10(4):e0115446. doi: 10.1371/journal.pone.0115446 (PMC4401726; doi:10.1371/journal.pone.0115446)
Supplement: S1 Appendix — (DOCX) [file pone.0115446.s011.docx]

**S1 Appendix.**

| **TOWN** | **Local Health Authority** |
| --- | --- |
| Harrogate | Harrogate Health Care |
| Shrewsbury | Shropshire health Authority |
| Lowestoft | Great Yarmouth James Pagent Healthcare NHS Trust |
| Mansfield | North Nottinghamshire Health |
| Southport | North Sefton Research Ethics Committee |
| Merthyr Tydfil | Awdurdod Lechyd Bro Taf Health Authority |
| Guildford | South West Surrey Local Research Ethics Committee |
| Burnley | East Lancashire Health Authority |
| NUL | North Staffordshire Health Authority |
| Exeter | Exeter Research Ethics Committee |
| Falkirk | Fife Health Board |
| Ipswich | East Suffolk Local Research Ethics Committee |
| Gloucester | Southmead Health Services |
| Carlisle | East Cumbria Local Research Ethics Committee |
| Dunfermline | Fife Health Board |
| Darlington | County Durham Local Research Ethics Committee |
| Ayr | Fife Health Board |
| Grimsby | South Humber Health Authority |
| Bedford | North Bedfordshire Health Authority |
| Wigan | Wigan & Leigh LREC |
| Scunthorpe | South Humber Health Authority |
| Hartlepool | Hartlepool Local Research Ethics Committee |
| Bristol | Southmead Health Services |
